# Vagus Nerve Stimulation Reduces Indomethacin-Induced Small Bowel Inflammation

**DOI:** 10.3389/fnins.2021.730407

**Published:** 2022-01-12

**Authors:** April S. Caravaca, Yaakov A. Levine, Anna Drake, Michael Eberhardson, Peder S. Olofsson

**Affiliations:** ^1^Laboratory of Immunobiology, Department of Medicine, Karolinska University Hospital, Solna, Sweden; ^2^MedTechLabs, BioClinicum, Stockholm Center for Bioelectronic Medicine, Karolinska University Hospital, Solna, Sweden; ^3^SetPoint Medical, Inc., Valencia, CA, United States; ^4^Institute of Bioelectronic Medicine, The Feinstein Institutes for Medical Research, New York, NY, United States; ^5^Department of Gastroenterology and Hepatology, University Hospital of Linköping, Linköping, Sweden; ^6^Department of Health, Medicine and Caring Sciences, Linköping University, Linköping, Sweden

**Keywords:** Crohn’s disease, inflammatory reflex, vagus nerve stimulation, small bowel, indomethacin, cholinergic anti-inflammatory pathway, bioelectronic medicine, inflammatory bowel disease

## Abstract

Crohn’s disease is a chronic, idiopathic condition characterized by intestinal inflammation and debilitating gastrointestinal symptomatology. Previous studies of inflammatory bowel disease (IBD), primarily in colitis, have shown reduced inflammation after electrical or pharmacological activation of the vagus nerve, but the scope and kinetics of this effect are incompletely understood. To investigate this, we studied the effect of electrical vagus nerve stimulation (VNS) in a rat model of indomethacin-induced small intestinal inflammation. 1 min of VNS significantly reduced small bowel total inflammatory lesion area [(mean ± SEM) sham: 124 ± 14 mm^2^, VNS: 62 ± 14 mm^2^, *p* = 0.002], intestinal peroxidation and chlorination rates, and intestinal and systemic pro-inflammatory cytokine levels as compared with sham-treated animals after 24 h following indomethacin administration. It was not known whether this observed reduction of inflammation after VNS in intestinal inflammation was mediated by direct innervation of the gut or if the signals are relayed through the spleen. To investigate this, we studied the VNS effect on the small bowel lesions of splenectomized rats and splenic nerve stimulation (SNS) in intact rats. We observed that VNS reduced small bowel inflammation also in splenectomized rats but SNS alone failed to significantly reduce small bowel lesion area. Interestingly, VNS significantly reduced small bowel lesion area for 48 h when indomethacin administration was delayed. Thus, 1 min of electrical activation of the vagus nerve reduced indomethacin-induced intestinal lesion area by a spleen-independent mechanism. The surprisingly long-lasting and spleen-independent effect of VNS on the intestinal response to indomethacin challenge has important implications on our understanding of neural control of intestinal inflammation and its potential translation to improved therapies for IBD.

## Introduction

Crohn’s disease and ulcerative colitis are two debilitating inflammatory bowel diseases (IBD) characterized by abdominal pain, weight loss, and frequent bowel movements. The hallmarks of Crohn’s disease are the involvement of the small bowel and the transmural inflammation in contrast to ulcerative colitis, which is limited to the colon and presents as a more superficial intestinal inflammation with bloody stools. IBD often affects patients relatively early in life, commonly with onset between 15 and 30 years of age (Dignass et al., 2012; Gomollón et al., 2017). Many of the therapeutic options involve systemic immunosuppressive drugs with potential long-term adverse effects such as infections and malignancies (Dignass et al., 2012; Torres et al., 2019). A significant fraction of patients do not respond adequately to available treatment and approximately half of the people affected by Crohn’s disease still require abdominal surgery (De Simone et al., 2021). Accordingly, there is an unmet need to improve effective treatment of Crohn’s disease while minimizing risks of serious side effects. This is particularly important in young patients who will require years of immunosuppressive maintenance treatment to keep the disease in remission. Interestingly, there is increasing evidence that neural reflexes regulate gut inflammation in health and disease, suggesting that targeting neural circuits is a potential therapeutic option in IBD (Goverse et al., 2016; Stakenborg and Boeckxstaens, 2021).

The *inflammatory reflex* is an example of a neural circuit that involves signaling in the vagus nerve that regulates both organ-specific and systemic immune activity (Borovikova et al., 2000; Rosas-Ballina et al., 2008; Eberhardson et al., 2020). In the motor arc of this circuit, the vagus nerve, the splenic nerve, choline acetyltransferase (ChAT^+^)-expressing T cells, and alpha7 nicotinic acetylcholine receptors (α7 nAChR) on innate immune cells are essential for inhibition of systemic release of TNF-α in inflammation (Wang et al., 2003; Rosas-Ballina et al., 2008, 2011; Olofsson et al., 2012; Tarnawski et al., 2018; Caravaca et al., 2019). The discovery and functional mapping of this reflex has supported progress to clinical trials of pharmacological or electrical activation of the inflammatory reflex for treatment of diseases characterized by excessive inflammation (Bonaz et al., 2016; Koopman et al., 2016; Consolim-Colombo et al., 2017; Olofsson and Bouton, 2019; Genovese et al., 2020; Yap et al., 2020).

In parallel to the spleen-dependent neural regulation of inflammation, a direct route to the gut has been proposed, in which efferent vagus nerve fibers functionally connect with the myenteric plexus in the intestinal wall (Berthoud et al., 1990, 1991), referred to as the *intestinal cholinergic anti-inflammatory pathway* (Goverse et al., 2016). This pathway also inhibits release of pro-inflammatory cytokines from macrophages and other cells by cholinergic signals (Rosas-Ballina et al., 2011; Tarnawski et al., 2018; Stakenborg and Boeckxstaens, 2021).

There are many different animal models of IBD that recapitulate certain aspects of Crohn’s disease and ulcerative colitis, but none fully capture the complexity of either clinical disease (Kiesler et al., 2001; Mizoguchi, 2012). An anti-inflammatory effect of vagus nerve stimulation (VNS) was observed in a number of studies of rodent colitis, such as DSS-, oxazolone-, and TNBS-colitis (Ghia et al., 2006; Bai et al., 2007; Snoek et al., 2010; Meregnani et al., 2011; Meroni et al., 2021) and others previously reviewed (Levine et al., 2018a). However, experimental data on VNS effect on lesions in the small bowel are largely lacking. This is important since nerve-mediated treatment mechanisms and effects may differ between anatomic regions and disease models (e.g., ulcerative colitis, Crohn’s disease, postoperative ileus) (Payne et al., 2019). Whether the reduction of inflammation observed after VNS in intestinal inflammation is mediated by direct innervation of the gut or if the signals are relayed through the spleen is not completely understood (Ji et al., 2014; Matteoli et al., 2014). Furthermore, the duration of VNS effects on reduction of intestinal inflammation is not known. A better understanding of the kinetics of VNS treatment on inflammatory lesions is important to inform both design of nerve stimulators and clinical trials in IBD (D’Haens et al., 2018; Tarnawski et al., 2018). Accordingly, we studied mechanism and kinetics of VNS in indomethacin-induced acute intestinal inflammation as a small bowel disease model.

## Materials and Methods

### Ethics Statement

This study and the experimental protocols were approved under #2010-008 by the Institutional Animal Care and Use Committee (IACUC; Manhasset, NY).

### Animals

Male Sprague Dawley rats (6–8 weeks old) were obtained from Taconic Farms, Inc., (Hudson, NY) and the study was conducted at The Feinstein Institutes for Medical Research (FIMR; Manhasset, NY). Animal care including room, cage and equipment sanitation conformed to the guidelines cited in the Guide for the Care and Use of Laboratory Animals, and the applicable standard operating procedures of FIMR. Animals were housed in a laboratory environment with temperatures ranging between 19.5 and 24.5°C and relative humidity between 30 and 70%. Automatic timers provided 12 h of light and 12 h of dark. Animals were allowed access *ad libitum* to Harlan Teklad Rodent Chow (Denver, CO) and fresh municipal tap water. This study was approved by FIMR’s Institutional Animal Care and Use Committee.

### Vagus Nerve Stimulation

The surgery and method used for VNS has been previously described (Olofsson et al., 2015; Kressel et al., 2020). The rats were anesthetized with a ketamine (100 mg/kg) and xylazine (10 mg/kg) intramuscular injection and secured in supine position. A ventral midline cervical incision was made between the mandible and sternum, and the subcutaneous tissue and salivary glands were bluntly separated and retracted laterally. The left cervical vagus nerve was isolated between the sternomastoid and sternohyoid muscles and secured with a custom-built bipolar cuff electrode (Microprobes, Gaithersburg, MD) with a silastic coated platinum-iridium wire lead. Sham stimulated rats were handled similarly but without electrical stimulation.

Stimulator (SetPoint Medical Corp., Valencia, CA)-driven charge-balanced biphasic pulses were generated using a bipolar current source and were capacitively isolated with > 1 uF ceramic capacitors on both electrode outputs. Rats were stimulated with a pulse waveform amplitude of 1 mA, 200 μs pulse width, and 50 μs inter-pulse-interval, previously demonstrated to activate vagus nerve fibers (Olofsson et al., 2015), for 60 s at 10 Hz. Following electrical stimulation, the electrode was removed, and the incision sutured with 4–0 braided silk.

### Splenic Nerve Stimulation

The surgery and method used for splenic nerve stimulation (SNS) has been previously described (Kressel et al., 2020). The rats were anesthetized and secured in a right lateral decubitus position. The spleen was carefully exposed through a 3 cm skin incision above the spleen, and the major splenic blood vessels were traced back to their convergence. The splenic nerve was isolated and suspended on a steel hook electrode (PlasticsOne, Roanoke, VA). Electrical SNS was performed with the same parameters as the VNS, 1 mA, 200 μs pulse width, and 50 μs inter-pulse-interval, for 60 s at 10 Hz. After stimulation, the hook electrode was removed, and the incision sutured in two layers with 4–0 braided silk. Sham SNS was performed by exposing the splenic nerve without electrode placement and electrical stimulation.

### Vagus Nerve Stimulation in Splenectomized Rats

The rats were anesthetized and secured in supine position. A bipolar cuff electrode was secured around the left carotid sheath as described above. The rats were gently repositioned to a right lateral decubitus position and the spleen exposed by a 3 cm incision on the left abdomen above the spleen. The major splenic blood vessels were isolated, ligated with 4–0 silk suture, cut distally to the suture, and the spleen removed. The incision was subsequently sutured in two layers with 4–0 braided silk, and electrical or sham VNS was performed as described above.

### Enteropathy Model

We used an enteropathy model known to produce lesions in the small intestine (Kent et al., 1969; Del Soldato et al., 1985; Yamada et al., 1993; Anthony et al., 2000). 0.5 h after VNS or sham procedure, the rats were injected subcutaneously on the back with 10 mg/kg indomethacin (5 mg/mL in 5% sodium bicarbonate) and returned to rack housing. For experiments evaluating the prolonged effect of VNS, indomethacin was injected either 0.5, 24, 48, or 72 h after the VNS or sham procedure. Evans Blue was administered intravenously under anesthesia (isoflurane; 3%) 0.5 h before euthanasia. Rats were euthanized by CO_2_ asphyxiation 24 h post induction of enteropathy. Blood was collected via cardiac puncture and clotted at room temperature for 1 h. The intestine from the proximal jejunum to the cecum was removed, cleaned, and formalin-fixed. The distal jejunum was assessed for histopathology.

For analysis of inflammatory mediators in the intestine and serum, Evans blue was withheld from a set of animals. In these animals, a standardized 2.54 cm section of the region between the distal jejunum and proximal half of ilium was cleaned, snap frozen in liquid nitrogen, and homogenized in a tissue protein extraction reagent (Pierce Biotechnology, Waltham, MA) containing a protease/phosphatase inhibitor cocktail (Roche, Basel, Switzerland). Lesion area in these rats was not quantitated.

### Assessment of Enteropathy and Serum Assays

Fixed intestines were flat-mounted, photographed, and the total lesion area was quantitated by a blinded scorer using digital morphometry (Scion Image; Scion Corporation or ImageJ; NIH). Photomicrographs were acquired of fixed jejunal samples that were embedded in paraffin and stained with hematoxylin and eosin. Chlorination and peroxidation activity were determined using EnzChek Myeloperoxidase Activity Assay Kit (Invitrogen, Waltham, MA) according to the manufacturer’s instructions. Intestinal IL-23 normalized to protein and serum HMGB1 concentrations were measured by western blot. Serum TNF was measured by ELISA (R&D Systems, Minneapolis, MN). Concentrations of select other mediators of intestinal inflammation were measured by a quantitative multiplexed electrochemiluminescence assay (Rat Discovery Kit, Meso Scale Discovery, Rockville, MD).

### Statistical Analysis

Differences in lesion areas between groups were analyzed by Student’s *t*-test or ANOVA with Bonferroni’s *post hoc* analysis. Hedges’ *g* was used to calculate effect size for Student’s *t*-test to measure magnitude of differences between groups^1^. Biomarker data was assumed to be normally distributed and Grubbs’ outlier test (α = 0.05) was used. Reduction from mean level of sham biomarkers in serum and tissue were analyzed with Student’s *t*-test. Data are shown as percentage of sham (mean ± SEM) unless otherwise specified and number of animals (n) are reported per group. *p* < 0.05 was considered significant. Statistical calculations were performed using the Prism 8 software (GraphPad software, San Diego, CA).

## Results

### Vagus Nerve Stimulation Reduced Small Bowel Inflammation in Indomethacin-Induced Enteropathy

**FIGURE 1:**
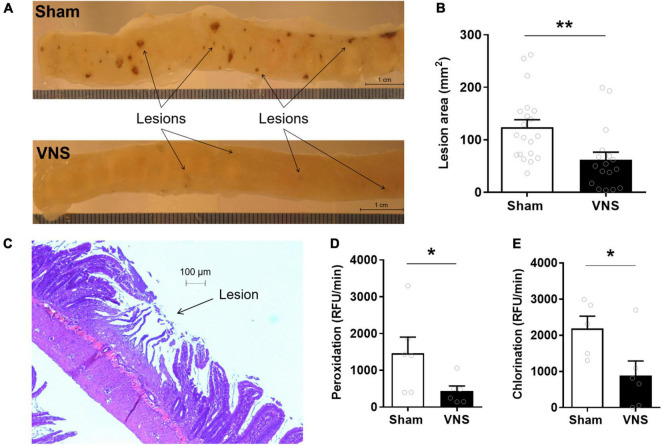
VNS reduced intestinal ulcerations in indomethacin-induced enteropathy. Rats were subjected to VNS- or sham-treatment followed by subcutaneous injection of 10 mg/kg indomethacin in 5% sodium bicarbonate. **(A)** Representative photograph of a jejunum section in sham and VNS-treated rats. **(B)** Quantification of total lesion area. **(C)** Photomicrograph of jejunum with visible mucosal lesion after staining with hematoxylin and eosin. **(D,E)** Myeloperoxidase activity in the small intestine as measured by **(D)** peroxidation and **(E)** chlorination assays. Data is presented as mean ± SEM. **p < 0.01 vs. sham **(B)**, *n* = 17–20 per group; *p < 0.05 vs. sham **(D,E)**, *n* = 6 per group.

Rats were subjected to cervical VNS or sham stimulation followed by subcutaneous injection of indomethacin. Small intestinal mucosal enteropathy was evaluated 24 h thereafter. Ulcers were observed almost exclusively in the distal portion of the small intestine (Figure 1A). Quantification by digital morphometric analysis showed a significantly smaller cumulative lesion area in the small intestine in VNS-treated animals as compared with sham ([mean lesion area ± SEM]: sham 124 ± 14 mm^2^, VNS: 62 ± 14 mm^2^, *p* < 0.002; effect size = 1.0) (Figure 1B). Microscopic analysis of ulcerated areas of the small intestine showed severe villus degradation (Figure 1C).

Myeloperoxidase (MPO) peroxidation activity, an established marker of neutrophil infiltration (Pozzoli et al., 2007), was significantly lower in VNS-treated as compared with sham-treated animals (Figures 1D,E). Peroxidation activity [Figure 1D; (RFU/min ± SEM) sham: 1,466 ± 439, VNS: 432 ± 142, *p* = 0.02; effect size = 1.3] and chlorination activity [Figure 1E; (RFU/min ± SEM) sham: 2,187 ± 342, VNS: 887 ± 402, *p* = 0.02; effect size = 1.3] were significantly reduced in VNS-treated compared with sham animals.

**FIGURE 2:**
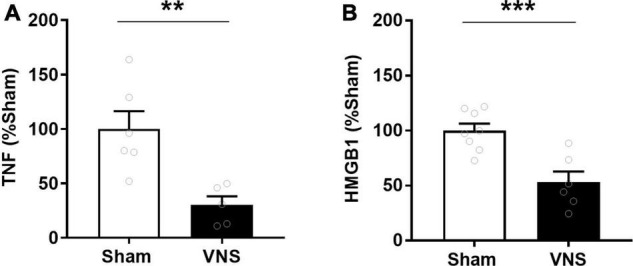
VNS reduced serum TNF in rats with indomethacin-induced enteropathy. Blood was collected and **(A)** serum TNF was measured by ELISA. *n* = 5–6 per group. **(B)** Serum HMGB1 was measured semiquantitatively by western blot and normalized to sham. *n* = 6–8 per group. Data is presented as mean ± SEM. **p < 0.01, ***p < 0.001 vs. sham.

**FIGURE 3:**
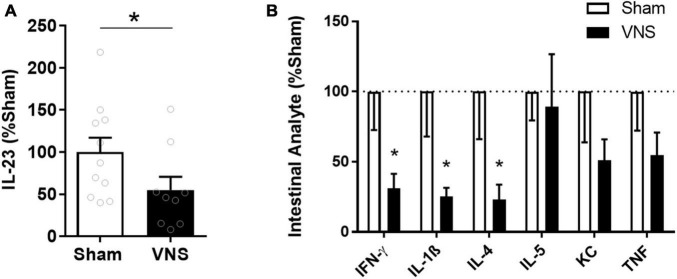
Intestinal levels of select cytokines in VNS- or sham-treated animals with indomethacin-induced enteropathy. **(A)** Semi-quantitative measurements by western blot of intestinal IL-23 were normalized to the mean sham level. *n* = 9–11 per group. **(B)** Levels of select cytokines were measured by quantitative multiplexed electrochemiluminescence assay and normalized to the mean sham level. *n* = 5–6 per group. Data is presented as mean ± SEM. *p < 0.05 vs. sham.

Serum TNF levels were significantly lower in VNS-treated rats compared with sham [(% of sham ± SEM) sham: 100 ± 16, VNS: 30 ± 8, *p* = 0.003; effect size = 2.2; Figure 2A]. Similarly, relative serum levels of the alarmin HMGB1 were significantly lower in VNS-treated animals as compared with sham animals [(% of sham ± SEM) sham: 100 ± 6, VNS: 53 ± 10, *p* = 0.0006; effect size = 2.3; Figure 2B and Supplementary Figure 1]. VNS-treated animals had significantly lower relative intestinal levels of IL-23 [(% of sham ± SEM) sham: 100 ± 17, VNS: 55 ± 16, *p* = 0.04; effect size = 0.9; Figure 3A and Supplementary Figure 1], IFN-γ, IL-1β, and IL-4 [(% of sham ± SEM) IFN-γ sham: 100 ± 27, VNS: 31 ± 11, *p* = 0.03; IL-1β sham: 100 ± 27, VNS: 31 ± 11, *p* = 0.03; IL-4 sham: 100 ± 34, VNS: 23 ± 11, *p* = 0.04; Figure 3B and Supplementary Table 1] as compared with sham. Mean relative levels of IL-5, KC, and TNF levels were lower, though not significant, in VNS vs. sham-treated animals [(% of sham ± SEM) IL-5 sham: 100 ± 21, VNS: 89 ± 37, *p* = 0.4; KC sham: 100 ± 36, VNS: 51 ± 15 *p* = 0.1; TNF sham: 100 ± 28, VNS: 55 ± 16, *p* = 0.1; Figure 3B and Supplementary Table 1]. IL-13-levels were at or below the lower limit of detection (2 pg/mL) and were not plotted.

### Vagus Nerve Stimulation Effect on Small Bowel Inflammation Independent of Spleen

**FIGURE 4:**
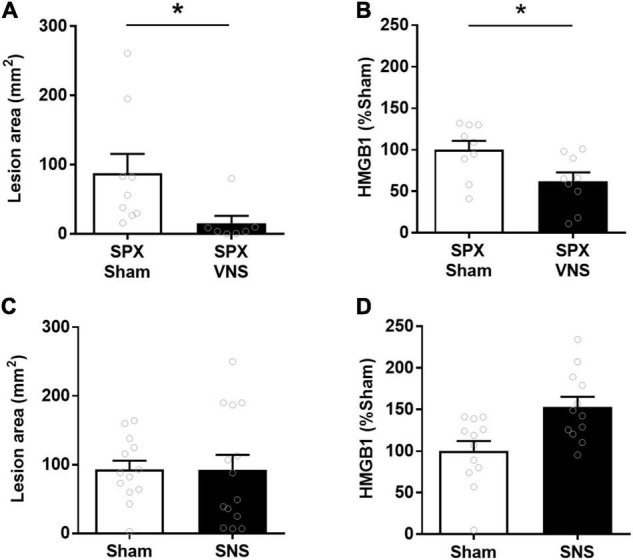
VNS reduced intestinal ulcerations in indomethacin-induced enteropathy independent of spleen. **(A,B)** Rats were subjected to splenectomy (SPX), followed by VNS or sham treatment and indomethacin administration. The small intestine lesion area was quantified at 24 h. Mean and SEM values are plotted (**A**, *n* = 7–9 per group; **B** 9 per group). **(C,D)** Rats were subjected to splenic nerve stimulation (SNS) or sham SNS surgery followed by indomethacin injection. The small intestine lesion area was quantified at 24 h. Serum HGMB1 was measured semiquantitatively by western blot and normalized to mean sham values (**C**, *n* = 13–14 per group; **D**, *n* = 12 per group). Data is presented as mean ± SEM. * *p* < 0.05 vs. sham.

In certain contexts, the effects of VNS on cytokines and inflammation require the splenic nerve and an intact spleen (Rosas-Ballina et al., 2008, 2011; Ji et al., 2014; Matteoli et al., 2014). To determine whether the spleen was required for the VNS effect in this model, a set of animals was splenectomized immediately before VNS and indomethacin administration. Lesions in the small intestine were quantified 24 h after injection. The cumulative lesion area in the small intestine was significantly lower in VNS-treated than in sham-treated animals also in the absence of the spleen [(mean lesion area ± SEM) sham: 88 ± 28 mm^2^, VNS: 15 ± 11 mm^2^, *p* = 0.02; effect size = 1.1; Figure 4A]. Furthermore, relative serum HMGB1 levels were significantly lower in VNS-treated as compared with sham-treated animals [(mean lesion area ± SEM) sham: 100 ± 11, VNS: 62 ± 11, *p* = 0.01; effect size 1.2; Figure 4B]. We first confirmed that SNS was effective in reducing endotoxin-induced serum TNF in this rat strain in an independent group of rats (Kressel et al., 2020). To determine whether SNS alone reduces indomethacin-induced ulcers in the small intestine, we performed electrical or sham SNS followed by indomethacin injection and measured intestinal lesion area 24 h later. We observed no significant difference in lesion area between animals treated with SNS or sham SNS [(lesion size in mm^2^ ± SEM) sham: 93 ± 13, SNS: 93 ± 22, *p* = 0.5; effect size = 0.01; Figure 4C]. In addition, SNS treated rats did not show significantly lower relative levels of serum HMGB1 levels [(% of sham ± SEM) sham: 100 ± 12, SNS: 153 ± 12, *p* > 0.9; effect size 1.3; Figure 4D]. Thus, SNS alone did not reduce small bowel lesion in indomethacin-induced enteropathy, nor was the spleen required for the protective effects of VNS in this model.

### Protection Against Small Bowel Inflammation Sustained 48 h After Vagus Nerve Stimulation

**FIGURE 5:**
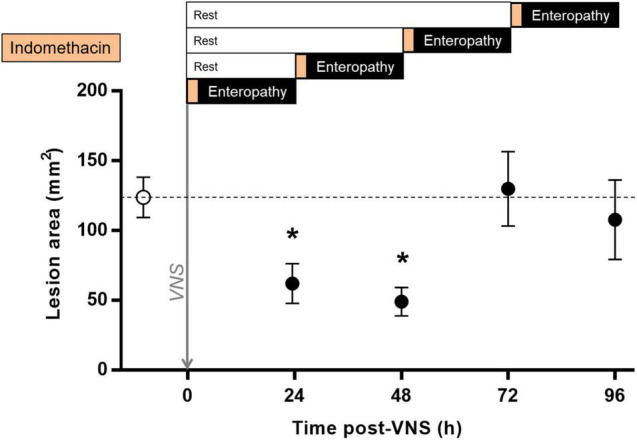
Sustained protection against indomethacin-induced intestinal ulcerations after VNS. Rats were subjected to VNS or sham surgery followed by a rest period of 0.5, 24, 48, or 72 h and subsequent subcutaneous injection of 10 mg/kg indomethacin in 5% sodium bicarbonate. Animals were injected systemically with Evans blue prior to euthanasia at 24 h after indomethacin injection. The small intestine was formalin-fixed, photographed and digitized. Total lesion area was quantified by a blinded scorer using Scion Image or ImageJ. Data is presented as mean ± SEM. *p < 0.05 vs. sham; *n* = 5–20 per group. Dotted line specifies the mean of the sham group.

In endotoxemia, a brief episode of stimulation, lasting seconds to minutes, has a sustained effect on the release of pro-inflammatory cytokines (Huston et al., 2007; Tarnawski et al., 2018). To investigate the duration of VNS effect on intestinal ulcer development in indomethacin-induced enteropathy, we performed VNS or sham surgery followed by a delayed induction of enteropathy, from 0.5 to 72 h. The area of intestinal ulceration was quantified at 24 h after indomethacin injection. We observed a significant decrease in intestinal lesion area when indomethacin was administered 0.5 or 24 h after VNS as compared with sham treatment [(lesion size in mm^2^ ± SEM) sham: 124 ± 14, 24 h post-VNS: 62 ± 14, *p* = 0.02; vs. 48 h post-VNS: 49 ± 10, *p* = 0.03; Figure 5]. In animals injected with indomethacin at 48 h or 72 h after VNS, there was no significant reduction in lesion area as compared with sham-treated animals [(lesion size in mm^2^ ± SEM) sham: 124 ± 14 vs. 72 h post-VNS: 130 ± 27, *p* > 0.9; vs. 96 h post-VNS: 108 ± 28, *p* > 0.9; Figure 5].

## Discussion

Here, we found that electrical stimulation of the vagus nerve reduced indomethacin-induced acute small bowel inflammation by a spleen-independent mechanism. Reduction of small bowel lesioning remained for 48 h after a single episode of VNS.

The findings here that electrical VNS reduced small bowel inflammation align with previous studies that show that signals in the vagus nerve regulate levels of pro-inflammatory cytokines and inflammation in a wide range of organs and scenarios (Steinberg et al., 2016). Reduction of the elevated levels of TNF that promote intestinal inflammation in Crohn’s disease has been a therapeutic success (Hanauer et al., 2006). However, after 1 year of anti-TNF treatment, more than half of the patients have stopped the treatment due to inadequate response, loss of response, or intolerance (Roda et al., 2016). Therefore, the additional evidence of vagus nerve regulation of intestinal lesion development provided here may be useful to support further development therapeutic modalities that target the neural regulation of inflammation.

The mechanism for vagus nerve regulation of intestinal inflammation is not fully understood. The vagus nerve directly innervates the gastrointestinal tract and is a key conduit for the bi-directional communication between the enteric nervous system and the central nervous system (Prechtl and Powley, 1990). The main neurotransmitter of the vagus nerve is acetylcholine, known not only to regulate visceral functions such as gastrointestinal motility, but also the release of pro-inflammatory cytokines from innate immune cells (Cailotto et al., 2014). Both afferent and efferent signals in the vagus nerve participate in the regulation of pro-inflammatory cytokines in inflammation (Olofsson et al., 2015). Data from murine models suggest that the reflex control of gut inflammation is mediated by the efferent vagus nerve and sympathetic nerve signals that culminate in regulating the activity of macrophages that reside in the viscera where vagus innervation is abundant (Luckey et al., 2003; Ji et al., 2014; Matteoli et al., 2014; Stakenborg and Boeckxstaens, 2021; Zhang et al., 2021).

Available data indicate that signals in the vagus nerve regulate gut inflammation by different routes, spleen-independent and spleen-dependent. One spleen-independent conduit involves the vagus nerve, efferent sympathetic nerves and myenteric efferent fibers that regulate macrophages in the intestinal wall (Luckey et al., 2003; Matteoli et al., 2014). Evidence of spleen-independent routes for vagus-nerve-mediated regulation of inflammation is also found in other visceral organs such as the pancreas: VNS reduced pancreatitis severity by a spleen-independent mechanism (Zhang et al., 2021).

A spleen-dependent route of vagus nerve regulation of inflammation is the inflammatory reflex, which involves efferent vagus neurons, the celiac ganglion, the splenic nerve, and the spleen (Ji et al., 2014; Kressel et al., 2020). These signals within the inflammatory reflex are propagated to immune cells in the spleen capable of biosynthesizing acetylcholine (Olofsson et al., 2012). Both spleen-dependent and spleen-independent neural regulation of inflammation may reduce macrophage accumulation and release of pro-inflammatory cytokines in the gut through activation of α7 nAChR, α4β2 nAChR or other cholinergic receptors (Wang et al., 2003; de Jonge et al., 2005; Rosas-Ballina et al., 2008, 2011; van der Zanden et al., 2009; Costantini et al., 2012). Of note, ChAT^+^ T cells, capable of acetylcholine biosynthesis, are found in the innervated Peyer’s patches of the small intestine of rodents (Vulchanova et al., 2007; Dhawan et al., 2016; Willemze et al., 2019) and it is possible that these cells partake in local regulation of immune cell activity as has been observed in spleen (Rosas-Ballina et al., 2011). Considering this, the finding here that the spleen is not essential for VNS-mediated reduction of disease intensity in indomethacin-induced small bowel inflammation is important. The data imply that for treatment of inflammatory lesions in IBD with involvement of the small intestine, targeting the cervical or gut-directed subdiaphragmatic vagus may be superior to targeting the splenic nerve or spleen.

The observation here that VNS reduced small intestinal inflammation for up to 48 h aligns with our previous finding that VNS reduced systemic TNF release in endotoxemia for up to 48 h (Huston et al., 2007; Tarnawski et al., 2018) and offers support for refining therapeutic stimulation protocols. The first published clinical trial using VNS for treatment of Crohn’s disease in biologic-naïve subjects was designed using the stimulation “duty cycle” (time on vs. time off) originally designed to treat epilepsy, stimulating up to 262 times per day (Bonaz et al., 2016). The findings here regarding the persistence of protection in the small bowel enabled a once per day VNS treatment in biologic-experienced subjects with Crohn’s disease (D’Haens et al., 2018). The growing mechanistic insights on regional differences in the neural regulation of inflammation between and within organs such as the colon, the small bowel, and other visceral organs, will be valuable for design of future IBD trials using bioelectronic medicine.

An interesting molecule in persistent inflammation is the alarmin HMGB1, which is involved in or associated with the pathogenesis of many inflammatory diseases and regulated by cholinergic signals (Huston et al., 2007; Andersson and Tracey, 2011). HMGB1 is a “late mediator” of inflammation, and excessive levels of HMGB1 may be involved in sustaining inflammation, with associated inflammatory tissue damage, organ dysfunction, and cognitive impairment (Andersson et al., 2000; Valdes-Ferrer et al., 2016; Bruck et al., 2020). Of note, anti-HMGB1 therapy was effective in significantly reducing disease severity even when administered days after onset of severe inflammation (Huston et al., 2007), and anti-HMGB1 antibodies and HMGB1-targeted sorption beads have a protective effect in experimental intestinal inflammation (Maeda et al., 2007; Yang et al., 2009; Ju et al., 2014). Based on that knowledge, it will be interesting to consider whether the link between VNS and reduced HMGB1 levels may help explain how VNS may be effective in reducing not only inflammation onset, but also ongoing inflammation (Huston et al., 2007; Bonaz et al., 2016). Accordingly, our observation here that VNS reduced serum levels of HMGB1 along with molecular markers of inflammation in the gut and small bowel lesion area is noteworthy. The potential role of HMGB1 in IBD is being further explored (Andersson and Tracey, 2011) and a pathogenetic role has been proposed for HMGB1-dependent TLR4 activation in indomethacin-induced small intestinal damage (Nadatani et al., 2012). The available data supports further consideration and study of the vagus nerve regulation of HMGB1, its potential as a biomarker and therapeutic target in IBD (Palone et al., 2016).

The current study, using a rat indomethacin-induced enteropathy model, provides evidence that electrical activation of the cervical vagus nerve reduces inflammatory lesions in the small bowel, a pathology that is commonly a critical part of the pathogenesis in Crohn’s disease (Liu et al., 2020). The rodent models of colitis that have previously been used to study vagus nerve and α7 nAChR-mediated regulation of disease severity are by many considered more relevant to ulcerative colitis than to Crohn’s disease (Snoek et al., 2010; Galitovskiy et al., 2011; Ghia et al., 2011; Ji et al., 2014). In humans, previous surgical vagotomy was associated with later development of Crohn’s disease, but not ulcerative colitis (Liu et al., 2020), suggesting that vagus nerve regulation of inflammation may be more important in small bowel inflammation than in colitis, although this remains to be comprehensively studied.

As described above, the first published study of VNS for the treatment of Crohn’s disease used high duty cycle nerve stimulation (Bonaz et al., 2016; Sinniger et al., 2020). While no major safety concerns were reported, higher frequency and intensity of electrical nerve stimulation consume more energy, thus requiring greater battery capacity for implanted stimulators. Furthering our understanding of the VNS parameter requirements for clinical benefit can therefore allow for optimization of device design and stimulation protocols. Longer periods between stimulations may also reduce patient discomfort both by limiting the stimulation-associated laryngeal muscle contraction and temporary voice changes as well as enabling smaller devices that are easier to implant and maintain (Levine et al., 2018b,c). Therefore, the observation that a short VNS period of 60 s was followed by a prolonged anti-inflammatory effect provides important information for the development of future clinical therapeutics.

## Conclusion

Electrical stimulation of the vagus nerve, but not of the splenic nerve, reduced indomethacin-induced small intestinal enteropathy for up to 2 days. The spleen was not required for the effect of VNS on small bowel lesion area. These observations are an important step toward better understanding of the mechanisms of vagus nerve regulation of inflammation in diseases with engagement of the small bowel.

## Data Availability Statement

The raw data supporting the conclusions of this article will be made available by the authors, without undue reservation.

## Ethics Statement

The animal study was reviewed and approved by the Feinstein Institutes for Medical Research Institutional Animal Care and Use Committee.

## Author Contributions

AC, AD, and YL planned and performed experiments. AC, AD, YL, ME, and PO analyzed and interpreted data. AC, YL, PO, and ME wrote the manuscript. All authors edited the manuscript.

## Conflict of Interest

YL is an employee of SetPoint Medical, Inc. AC and AD were employees of SetPoint Medical, Inc. at the time of data collection. Aside from co-author affiliation, this funder was not involved in the study design, data analyses, data interpretation, and the writing of the report. ME has received honoraria for lectures and consultancy from AbbVie, Merck (MSD), Takeda, Ferring, Orion Pharma, Otsuka, Tillotts, Novartis, Pfizer, and Janssen, received research funding from AbbVie and MSD, and has been a former shareholder of Emune AB. These funders were not involved in the study design, data analyses, data interpretation, and the writing of the report. PO has received honoraria for lectures from Ferring and Janssen and is a shareholder of Emune AB. AC and PO were supported by MedTechLabs, Stockholm. These funders were not involved in the study design, data analyses, data interpretation, and the writing of the report.

## Publisher’s Note

All claims expressed in this article are solely those of the authors and do not necessarily represent those of their affiliated organizations, or those of the publisher, the editors and the reviewers. Any product that may be evaluated in this article, or claim that may be made by its manufacturer, is not guaranteed or endorsed by the publisher.

## Footnotes

1
https://www.socscistatistics.com/effectsize

## Supplementary Material

Supplementary Figure 1 VNS reduced IL-23 and HMGB1 in small bowel inflammation. Representative densitometry from western blot of **(A)** serum HMGB1 and **(B)** intestinal IL-23. Mean quantified integrated intensity (IKK count) of HMGB1 in the sham group was 0.69. Mean quantified integrated intensity (IKK count) of IL-23/Actin in the sham group was 0.25.

Supplementary Table 1 VNS reduced levels of select cytokines in indomethacin-induced enteropathy. Levels of select cytokines were measured by quantitative multiplexed electrochemiluminescence assay and normalized to the mean sham level. Data is presented as mean ± SEM. * *p* < 0.05 vs. sham.
