# Supplementary figures and images for: Vagus Nerve Stimulation Reduces Indomethacin-Induced Small Bowel Inflammation
Source: Front Neurosci. 2022 Jan 12;15:730407. doi: 10.3389/fnins.2021.730407 (PMC8789651; doi:10.3389/fnins.2021.730407)

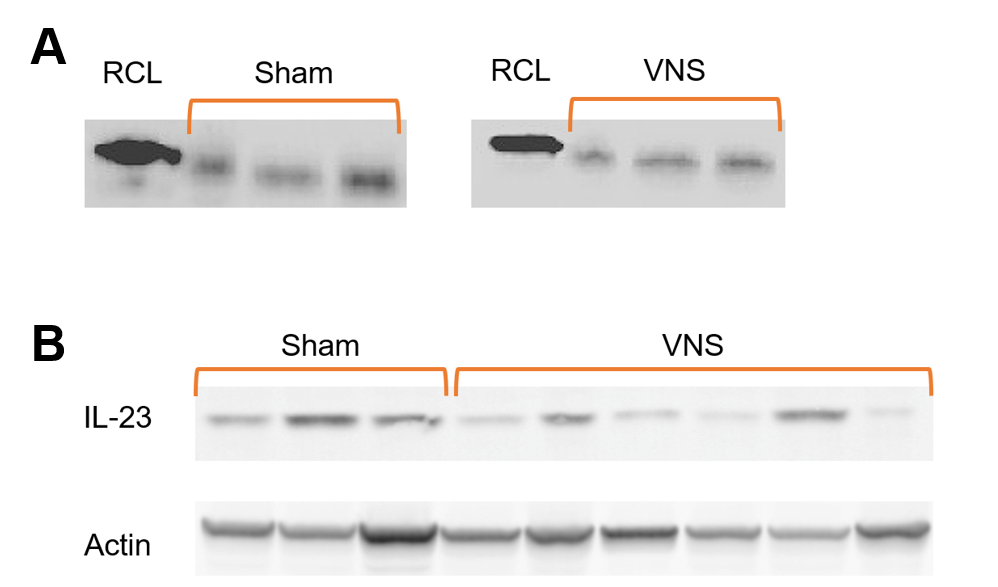

Supplement: Supplementary Figure 1 — VNS reduced IL-23 and HMGB1 in small bowel inflammation. Representative densitometry from western blot of (A) serum HMGB1 and (B) intestinal IL-23. Mean quantified integrated intensity (IKK count) of HMGB1 in the sham group was 0.69. Mean quantified integrated intensity (IKK count) of IL-23/Actin in the sham group was 0.25. [file Image_1.TIF]
