# Supplementary material for: Vagus Nerve Stimulation Reduces Indomethacin-Induced Small Bowel Inflammation
Source: Front Neurosci. 2022 Jan 12;15:730407. doi: 10.3389/fnins.2021.730407 (PMC8789651; doi:10.3389/fnins.2021.730407)
Supplement: Supplementary Table 1 — VNS reduced levels of select cytokines in indomethacin-induced enteropathy. Levels of select cytokines were measured by quantitative multiplexed electrochemiluminescence assay and normalized to the mean sham level. Data is presented as mean ± SEM. * p < 0.05 vs. sham. [file Table_1.pdf]

SUPPLEMENTARY TABLE 1

Analyte	Sham		VNS		*t*-test
	Mean (pg/mg)	SEM	Mean (pg/mg)	SEM	
IFN-γ	19.4	5.3	6.0	2.0	p = 0.03 *
IL-1β	685.5	219.7	172.2	42.8	p = 0.03 *
IL-4	3.1	1.0	0.7	0.3	p = 0.04 *
IL-5	38.0	7.8	33.7	14.2	p = 0.4
KC	1902.7	688.0	969.2	285.0	p = 0.1
TNF	75.0	20.9	41.0	12.0	p = 0.1
